# Comparative effects of transcranial direct and alternating current stimulation combined with cognitive-motor dual-task training on functional and cognitive recovery in stroke survivors

**DOI:** 10.3389/fneur.2026.1720429

**Published:** 2026-02-06

**Authors:** Yutong Fu, Wenli Wang, Qianxi Yan, Jin Song, YanMei Li, Chang Zhu, Siaw Chui Chai, Ponnusamy Subramaniam, Liqing Yao, Devinder Kaur Ajit Singh

**Affiliations:** 1 Department of Rehabilitation Medicine, The Second Affiliated Hospital of Kunming Medical University, Kunming, China; 2 Center for Healthy Ageing and Wellness (H-CARE), Faculty of Health Sciences, Universiti Kebangsaan Malaysia, Kuala Lumpur, Malaysia; 3 Centre for Rehabilitation and Special Needs Studies (iCaRehab), Faculty of Health Sciences, Universiti Kebangsaan Malaysia, Kuala Lumpur, Malaysia

**Keywords:** cognitive-motor dual-task, function, stroke, transcranial alternating current stimulation, transcranial direct current stimulation

## Abstract

**Aim:**

This study aimed to determine whether dual-target anodal transcranial direct current stimulation (tDCS) or transcranial alternating current stimulation (tACS) applied over the primary motor cortex (M1) and left dorsolateral prefrontal cortex (DLPFC), when combined with cognitive-motor dual-task training (CMDT), could enhance cognitive, physical, and mood outcomes in stroke survivors.

**Methods:**

A single-blind, three-arm randomized controlled trial was conducted involving 72 stroke survivors. Participants were randomized to receive 15 sessions of dual-site (M1 + DLPFC) anodal tDCS, tACS, or sham stimulation, all administered concurrently with CMDT. Primary outcomes included cognitive performance (Visual Cognitive Assessment Test, VCAT) and physical function (Fugl-Meyer Lower Limb, FMA-LE). Secondary outcomes comprised the Trail-Making Test (TMT-A/B), Berg Balance Scale (BBS), Timed Up and Go (TUG) under single and dual-task conditions, and the Hamilton Depression Scale (HAMD). Data were analyzed using ANOVA with Bonferroni-adjusted pairwise comparisons.

**Results:**

Significant group × time interactions were observed across multiple functional domains. The tDCS + CMDT group showed superior improvements in FMA-LE scores vs. both tACS + CMDT and sham + CMDT (post-hoc *p* < 0.05 each), but in BBS scores only vs. sham + CMDT (p < 0.05). Additionally, tDCS significantly enhanced VCAT performance compared to sham (*p* < 0.05). Both tDCS and tACS groups produced comparable and significantly greater improvements than sham in TUG-Dual performance and HAMD reduction (*p* < 0.001).

**Conclusion:**

Dual-site anodal tDCS and tACS combined with CMDT represent safe and effective neuromodulatory strategies for stroke rehabilitation. While tDCS confers greater benefits in promoting local motor plasticity and global cognitive gains, tACS achieves functionally equivalent improvements in network-dependent tasks such as dual-task performance and mood regulation.

**Significance:**

These findings provide novel, evidence-based guidance for tailoring non-invasive brain stimulation (NIBS) modalities to specific neurorehabilitation goals, highlighting that combining tDCS or tACS with CMDT can enhance neuroplasticity and translate into meaningful functional recovery in stroke survivors.

**Clinical trial registration:**

Clinical Trial Registry (ChiCTR2400092849).

## 1 Introduction

Stroke is a major global health challenge, ranking as the second leading cause of death and the third leading cause of death and disability combined (1, 2). With over 100 million people worldwide having experienced a stroke (3), survivors frequently face persistent impairments in motor, cognitive, and multiple functional domains (3–8), particularly in low- and middle-income regions (9). Projected increases in incidence due to population aging are expected to drive annual stroke-related medical costs substantially higher in coming decades (10).

Executive dysfunction is common post-stroke, affecting a substantial proportion of survivors and often involving deficits in processing speed, planning, working memory, attention, and inhibitory control (11). These impairments critically influence independence in daily activities, social engagement, and quality of life (12), contributing to risks such as reduced mobility and falls (13, 14).

Stroke survivors commonly exhibit greater interference during dual-task conditions, such as walking while performing cognitive tasks, resulting in decrements in gait speed, adaptability, and cognitive performance compared to single-task execution (15, 16). This elevated dual-task cost heightens fall risk and restricts participation in real-world activities (17). Traditional rehabilitation approaches often address motor and cognitive deficits separately, yet everyday tasks typically require integrated cognitive-motor processing, known as cognitive-motor dual-task (CMDT) (16). Difficulties with activities of daily living (ADLs) represent a primary concern for many survivors (18), underscoring the need for ecologically valid interventions.

Emerging evidence supports CMDT as an effective rehabilitation strategy that simultaneously engages executive functions and motor demands, promoting better transfer to functional contexts and improvements in flexibility and self-care (17, 19, 20).

To enhance neuroplasticity and amplify training effects, non-invasive brain stimulation techniques like transcranial direct current stimulation (tDCS) and transcranial alternating current stimulation (tACS) show promise (21, 22). tDCS modulates cortical excitability (23), while tACS entrains neural oscillations in a frequency-specific manner (24). Targeting regions such as the primary motor cortex (M1) for motor recovery and dorsolateral prefrontal cortex (DLPFC) for executive functions (25–27), combined stimulation with CMDT may optimize resource allocation and functional outcomes through primed neuroplasticity (28).

Therefore, in this study, we aimed to investigate the effects of tDCS and tACS combined with CMDT training on physical, cognitive and ADL functions.

## 2 Materials and methods

### 2.1 Participants

This study included 72 (44 males, 25 females; age: 56.18 ± 7.73 vs. 56.78 ± 9.22 vs. 56.04 ± 8.86 years in tDCS vs. tACS vs. sham groups, each combined with CMDT) stroke survivors who were recruited from the Rehabilitation Department of the Second Affiliated Hospital of Kunming Medical University Hospital between 1 December 2024 and 1 September 2025. All participants provided written informed consent, which was properly documented and witnessed by either medical staff or family members. According G*Power 3.1.9.2 software was used to calculate the minimum sample size of the research objects. Using a medium effect size of 0.25, a power of 0.95, and anα value of 0.05, it is calculated that the total number of samples in the study should be at least 66. Considering that there may be 10% sample loss, therefore, the number of participants in this study should be at least 72. We initially used stratified block randomisation (block size 6, 1:1:1) within age-and-sex strata, generated by CREATE A RANDOMISATION LIST and implemented with sequentially numbered, opaque, sealed envelopes. After obtaining informed consent, the researcher notified an independent staff member responsible for randomization, who was not involved in participant contact or outcome assessment and had no access to clinical data. The assigned group was then communicated via text message to the technician responsible for programming the tES device. The participants, evaluators and therapists were blinded to group assignment.

Inclusion criteria were: (1) diagnosis of ischemic or hemorrhagic stroke confirmed by neuroimaging, middle cerebral artery region, (2) stroke onset within 7 days-6 months from the start of the intervention, (3) ability to follow instructions and provide informed consent, (4) MoCA score between 7 and 26, (5) able walk at least 3 m independently with assistive devices, (6) age between 40 and 75 years old, (7) neuroimaging-confirmed (MRI/CT) stroke located in the Middle Cerebral Artery (MCA) distribution.

Exclusion criteria included: (1) neurological disorder other than stroke, (2) severe musculoskeletal disease, (3) severe speech impairment, (4) severe pain during exercise or rest, (5) mental disease, (6) cardiovascular diseases that affect rehabilitation, such as heart failure, (7) severe visual or hearing impairment, (8) any contraindications to tDCS such as skull defects, intracranial implantation of metal.

### 2.2 Procedures

**Figure 1:**
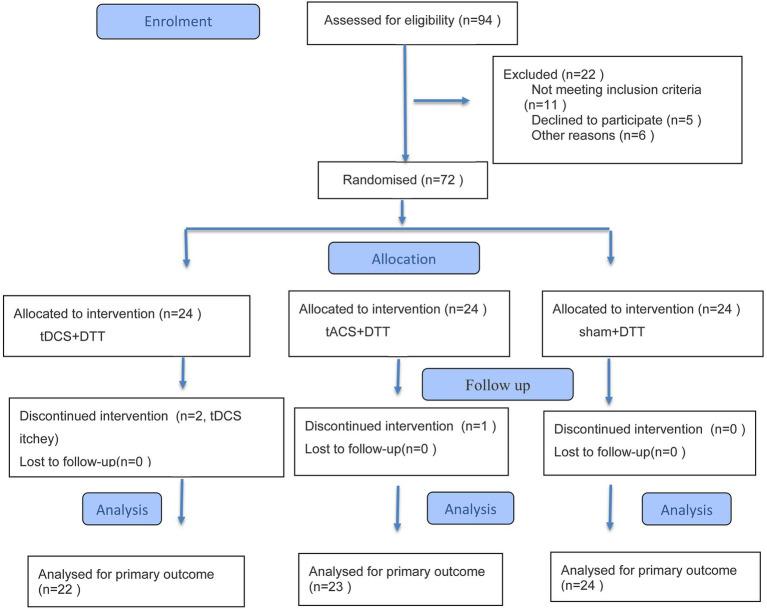
Flow diagram in this study.

In this randomized, blinded, sham-controlled parallel study, participants with subacute stroke received five sessions per week for 3 weeks, totaling 15 intervention sessions. Participants were randomly assigned to receive either dual-anodal tDCS targeting two cortical sites in combination with CMDT, or sham tDCS in addition to CMDT. Active tDCS + CMDT group (*n* = 24), tACS + CMDT group (*n* = 24), received dual-anodal tDCS targeting the primary motor cortex (M1) and dorsolateral prefrontal cortex (DLPFC), combined with CMDT. While, sham + CMDT group (*n* = 24), received sham stimulation with an identical CMDT protocol (Figure 1).

### 2.3 tDCS and tACS protocol

Both tDCS and tACS are forms of transcranial electrical stimulation (tES). tES was administered using the stimulator with two anodal electrodes (5 × 7 cm gelatin sponge) positioned over the affected M1 and left DLPFC, localized via the 10–20 EEG system (M1: C3/C4 depending on the affected hemisphere; DLPFC: F3). The cathodal electrode (5 × 7 cm) was placed over the contralateral supraorbital area (FP2) to minimize off-target effects.

Stimulation parameters included: Current intensity: 2 mA, 40HZ tACS-DLPFC [based on based on our team’s previous research findings (25)]. Duration: 20 min/session. Frequency: 5 sessions/week for 3 weeks, in total of 15 sessions. The sham group received identical electrode placement with 30-s ramp-up/down phases and no sustained current, ensuring participant blinding.

All participants were closely monitored for any adverse effects related to tDCS or tACS during the intervention. Adverse events, including skin irritation, dizziness, or discomfort, were recorded and assessed in accordance with established safety protocols. Any serious adverse events were reported to the ethics committee immediately.

### 2.4 CMDT

CMDT sessions involved simultaneous motor-cognitive challenges, such as: walking while performing serial subtraction (100–3, 97–3). Tasks were adapted weekly based on performance (increasing cognitive load or motor complexity) to maintain a 70–80% success rate. Each session lasted 30–40 min, delivered 5 days/week for 3 weeks, in total of 15 sessions.

Regular rehabilitation training: Participants receive single task based physical, occupational or speech therapy conducted by licensed therapist once a day, 5 days/week for 3 weeks, in total of 15 sessions. Participants also received standard, single-task-based rehabilitation tailored to their individual assessment outcomes. Depending on clinical needs, they underwent physical, occupational, or speech therapy delivered by licensed therapists once daily, 5 days per week, for a total of 15 sessions over 3 weeks.

Adherence and fidelity were rigorously monitored: All training sessions were supervised by licensed physical therapists specifically trained in the study protocol. Only one participant withdrew from CMDT during the study. Fidelity was ensured through standardized session checklists, confirming consistent task delivery, timing, and progression, with high compliance and no major protocol deviations reported.

### 2.5 Screening and outcome measures

The Montreal Cognitive Assessment (MoCA) (29) was used as a screening tool to assess cognitive function for determining eligibility based on the inclusion criteria.

#### Outcome measures

The Visual Cognitive Assessment Test (VCAT) was used to assess cognitive performance as an outcome measure (30). In Chinese version (31), the VCAT and its subdomains demonstrated both good construct validity and internal consistency (*α* = 0.577) (32). The sensitivity of TMT-B (87%) is superior to that of TMT-A (69%) and the specificity is similar (90% vs. 88%, respectively) (33). This test has a high retest reliability result of 0.79 for TMT-A and 0.89 for TMT-B (33). TUG test–retest reliability is 0.96 (32). FMA-LE is the primary motor outcome and the retest reliability rating scale is ≥0.95 (34). High correlation values are obtained between FM and the BI (r = 0.9167; *p<*0.0001) (35). BBS interobserver reliability is excellent, both for single items (0.84–0.98, *p* < 0.001) and for the total score (ICC = 0.95; 95% confidence interval = 0.910–0.975) (28). In the Chinese version (36), the retest reliability of the total score of MBI is ICC = 0.909–0.991, and the inter-rater reliability of the total score is ICC = 0.880 ~ 0.992. In the Chinese version (37), the internal consistency reliability is good, Cronbach’s *α* = 0.836, high correlation values are obtained between HAMD and the GAS (*r* = 0.84), and the structural validity is also good.

### 2.6 Statistical analysis

Statistical analysis was performed using SPSS 27.0. Behavioral data in this study refers to clinical scores evaluating cognitive (VCAT, TMT-A/B), physical (TUG, FMA-LE, BBS, MBI), and psychological (HAMD) functions. Descriptive statistics were generated for all variables. Baseline comparability among the three groups was assessed using one-way analysis of variance (ANOVA) for continuous, normally distributed data, the Kruskal-Wallis *H* test for non-normally distributed data, and Chi-squared tests for categorical data. To evaluate intervention effects, a repeated measures ANOVA was conducted with time (Week 0 vs. Week 3) as the within-subject factor and group (tDCS + CMDT, tACS + CMDT, Sham + CMDT) as the between-subject factor. Post-hoc analyses were performed using Bonferroni-corrected pairwise comparisons to identify specific differences between groups at Week 3. Significance for all post-hoc multiple comparisons was determined using the Bonferroni correction, and the results are reported as adjusted *p*-values. The significance level was set at *α* = 0.05.

### 2.7 Adverse events

In the tDCS group, two participants reported mild to moderate headaches. As these symptoms persisted beyond two consecutive sessions, both participants discontinued further tDCS in accordance with the trial’s safety protocol. No participants in the tACS group or Sham group discontinued treatment due to adverse effects. No participants withdrew or dropped out during the CMDT training or the overall intervention period.

## 3 Results

### 3.1 Baseline characteristics

Baseline characteristics of participants including age, MoCA scores, stroke onset time, gender, affected side, stroke type, TUG single-task and dual-task walking times, VCAT, MBI, FMA-LE, BBS, and TMT-A/B are summarized in Table 1. One-way ANOVA and chi-squared tests revealed no significant differences among the tDCS + CMDT, tACS + CMDT, and Sham + CMDT groups on these variables except subgroup onset (<3 m) (*p* > 0.05), indicating good baseline comparability.

**Table 1:** Sociodemographic and clinical data of participants.

Sociodemographic and clinical data	tDCS + CMDT *n* = 22	tACS + CMDT *n* = 23	Sham + CMDT *n* = 24	*p*
Sociodemographic				
Age (years)	56.18 ± 7.73	56.78 ± 9.22	56.04 ± 8.86	0.95
Onset (months)	4.19 ± 1.16	3.79 ± 1.51	3.15 ± 1.74	0.06
≤3 m/3-6 m	4/18	10/13	14/10	0.02
Gender (male/female)	12/10	14/9	18/6	0.53
Affected side (left/right)	9/13	12/11	13/11	0.63
Stroke type (ischemic/hemorrhage)	12/10	14/9	14/10	0.913
Walking aids number (N)	11	9	9	0.65
Cognitive function				
Moca (score)	16.59 ± 5.43	17.00 ± 5.38	19.54 ± 4.67	0.11
VCAT (score)	20.81 ± 4.38	19.13 ± 6.12	19.62 ± 4.27	0.51
TMT-A (seconds)	82.23 ± 40.68	72.71 ± 30.91	83.71 ± 37.62	0.54
TMT-B (seconds)	125.17 ± 63.98	106.32 ± 50.05	118.21 ± 39.99	0.47
Physical function				
TUG-single walking time (seconds)	35.61 ± 17.86	32.78 ± 12.20	35.03 ± 17.04	0.82
TUG-CMDT (walking and 100–3) time (seconds)	48.00 ± 20.58	43.43 ± 7.33	48.42 ± 20.85	0.57
FMA-LE (score)	22.59 ± 7.26	24.90 ± 5.62	24.41 ± 6.64	0.46
BBS (score)	42.00 ± 9.39	45.04 ± 7.49	44.33 ± 7.35	0.43
ADL				
MBI (score)	84.31 ± 14.74	85.45 ± 12.14	80.00 ± 12.68	0.33
Psychology				
HAMD (score)	8.77 ± 3.02	8.40 ± 2.17	9.87 ± 1.94	0.10

Data which were normal were shown as mean (standard deviation) with a *p*-value for ANOVA except for affected side (left/right), gender (male/female) with *p*-value for chi-squared test. Statistically significant, *p* < 0.05. BBS, Befg Balance Scale; MBI, Modified barthel index; HAMD, Hamilton’s Depression Scale; FMA-LE, Fulg-Meyer lower limb; TMT, trail making test; CMDT, cognitive-motor dual task; TUG, timed up and go test; VCAT, visual cognitive assessment test. MOCA, Montreal Cognitive Assessment.

### 3.2 Primary outcomes

Repeated measures ANOVA revealed a significant interaction effect between time and group for VCAT scores (*p* = 0.016). Bonferroni post-hoc comparisons showed that the tDCS + CMDT group achieved significantly higher scores compared to the Sham + CMDT group (*p* < 0.05). No significant differences were found between the tDCS + CMDT and tACS + CMDT groups or between the tACS + CMDT and Sham + CMDT groups (*p* > 0.05).

There was a significant interaction effect between time and group for FMA-LE scores (*p* < 0.001). Post-hoc analysis indicated that the tDCS + CMDT group had significantly higher scores than both the Sham + CMDT group (*p* < 0.05) and the tACS + CMDT group (*p* < 0.05). The comparison between the tACS + CMDT and Sham + CMDT groups was not significant (*p* > 0.05) (Table 2).

**Table 2:** Main effect of time, group, and time–group interaction of the interventions on the physical and cognitive functions.

Parameters	Study group			Analysis of covariance (*p*-value)		
	tDCS + CMDT	tACS + CMDT	Sham + CMDT	Time (*ηp*^2^)	Group (*ηp*^2^)	Interaction (*ηp*^2^)
	(mean ± SD)	(mean ± SD)	(mean ± SD)			
Cognitive measure						
VCAT (score)						
Week 0	20.81 ± 4.38	19.13 ± 6.12	19.62 ± 4.27	<0.001 (0.64)	0.31 (0.03)	**0.016** (0.05)
Week 3	25.27 ± 3.14	24.09 ± 3.75	22.83 ± 4.57			
TMT-A performance (seconds)						
Week 0	82.23 ± 40.68	72.71 ± 30.91	83.71 ± 37.62	<0.001 (0.52)	0.06 (0.08)	0.001 (0.18)
Week 3	57.15 ± 24.99	54.36 ± 24.46	76.82 ± 30.27			
MBI (score)						
Week 0	84.31 ± 14.74	85.45 ± 12.14	80.00 ± 12.68	<0.001 (0.44)	0.42 (0.02)	0.30 (0.03)
Week 3	87.72 ± 113.33	88.18 ± 11.70	84.58 ± 10.92			
HAMD (score)						
Week 0	8.77 ± 3.02	8.40 ± 2.17	9.87 ± 1.94	<0.001 (0.74)	**<0.001** (0.29)	**<0.001** (0.40)
Week 3	3.22 ± 2.22	3.90 ± 3.30	8.58 ± 2.91			
Physical function						
FMA-LE (score)						
Week 0	22.59 ± 7.26	24.90 ± 5.62	24.41 ± 6.64	<0.001 (0.67)	0.76 (0.00)	**<0.001** (0.45)
Week 3	30.00 ± 5.19	27.72 ± 4.78	26.04 ± 6.13			
BBS (score)						
Week 0	42.00 ± 9.39	45.04 ± 7.49	44.33 ± 7.35	<0.001 (0.33)	0.91 (0.00)	0.007 (0.14)
Week 3	49.81 ± 4.18	47.72 ± 7.24	46.79 ± 6.22			
TUG-single walking time (seconds)						
Week 0	35.61 ± 17.86	32.78 ± 12.20	35.03 ± 17.04	<0.001 (0.54)	0.77 (0.00)	<0.001 (0.32)
Week 3	21.85 ± 11.09	28.67 ± 10.51	31.33 ± 15.69			
TUG-CMDT (walking and 100–3) (seconds)						
Week 0	48.00 ± 20.58	43.43 ± 7.33	48.42 ± 20.85	<0.001 (0.59)	0.15 (0.05)	0.002 (0.17)
Week 3	28.50 ± 10.32	26.70 ± 9.20	41.55 ± 18.97			

Bold values indicate statistical significance at the *p* < 0.05 level.

### 3.3 Secondary outcomes

Significant interaction effects were observed for both TMT-A (*p* = 0.001) and TMT-B (*p* = 0.04) performance. However, following Bonferroni correction, post-hoc pairwise comparisons revealed no significant differences between any of the three groups for TMT-A or TMT-B (*p* > 0.05).

A significant interaction effect was found for BBS scores (*p* = 0.007). Bonferroni post-hoc comparisons showed that the tDCS + CMDT group achieved significantly greater scores than the Sham + CMDT group (*p* < 0.05). There were no significant differences between the tDCS + CMDT and tACS + CMDT groups or between the tACS + CMDT and Sham + CMDT groups (*p* > 0.05).

A significant interaction effect for TUG-dual task performance (*p* = 0.002). Post-hoc analysis revealed that both the tDCS + CMDT group and the tACS + CMDT group (*p* < 0.05) completed the task significantly faster than the Sham + CMDT group. No significant difference was found between the two active stimulation groups (*p* > 0.05).

A highly significant interaction effect was revealed for HAMD scores (*p* < 0.001). Post-hoc comparisons indicated that scores were significantly lower in both the tDCS + CMDT group and the tACS + CMDT group (p < 0.001), relative to the Sham + CMDT group. No significant difference was observed between the tDCS + CMDT and tACS + CMDT groups (*p* > 0.05).

While a significant interaction effect was observed for TUG-single task performance (*p* < 0.001), subsequent Bonferroni post-hoc comparisons showed no significant pairwise differences between any groups (*p* > 0.05). No significant interaction effect between time and group was found for MBI scores (*p* > 0.05) in Table 3 and Figure 1.

**Table 3:** Post hoc multiple comparison among three groups.

Variable	Group (I)	Group (J)	Mean Difference (I–J)	SE	Adjusted *p*-value	95% CI	Effect size (Cohen’s *d*)
Cognitive							
VCAT (score)	tDCS	sham	3.33	1.28	**<0.05**	0.23–6.44	0.78
	tDCS	tACS	1.14	1.12	0.93	−1.61	0.31
	tACS	sham	0.34	1.27	1.00	−2.72–3.41	0.08
TMT-A (seconds)	tDCS	sham	−14.07	13.75	1.00	−47.34–19.19	−0.31
	tDCS	tACS	15.69	13.97	1.00	−18.00–49.38	0.34
	tACS	sham	−29.76	13.56	0.09	−62.59–3.05	−0.66
TMT-B (seconds)	tDCS	sham	−2.95	11.72	1.00	−31.76–25.85	−0.08
	tDCS	tACS	17.86	11.98	0.42	−11.58–47.28	0.45
	tACS	sham	−20.81	11.73	0.24	−49.62–8.00	−0.54
Physical							
TUG-single task (seconds)	tDCS	sham	−4.46	1.99	0.11	−9.36–0.44	−0.68
	tDCS	tACS	−2.00	2.01	1.00	−6.94–2.94	−0.3
	tACS	sham	−2.46	1.99	1.00	−7.34–2.43	−0.37
TUG-dual task (seconds)	tDCS	sham	−13.07	3.61	**<0.05**	−21.92−−4.20	1.09
	tDCS	tACS	−1.91	3.66	1.00	−10.88–7.06	−0.16
	tACS	sham	−11.16	3.59	**<0.05**	−19.95−−2.36	0.94
FMA-LE (score)	tDCS	sham	3.78	1.01	**<0.05**	1.29–6.27	1.13
	tDCS	tACS	2.46	1.02	**<0.05**	0.15–4.76	0.73
	tACS	sham	1.33	1.00	0.42	−1.13–3.78	0.4
BBS (score)	tDCS	sham	3.15	1.08	**<0.0**5	0.51–5.79	0.88
	tDCS	tACS	2.44	1.10	0.09	−0.22–5.11	0.67
	tACS	sham	0.71	1.07	1.00	−1.90–3.31	0.2
Psychology							
HAMD (score)	tDCS	sham	−5.36	1.05	**<0.001**	−7.91−−2.81	1.54
	tDCS	tACS	0.09	1.06	1.00	−2.51–2.69	0.03
	tACS	sham	−5.44	1.04	**<0.001**	−7.99−−2.90	1.58

tDCS, transcranial direct current stimulation; tACS, transcranial alternating current stimulation; and Sham, Sham control group. *p*-values were derived from repeated measures ANOVA and adjusted using Bonferroni correction for multiple comparisons. Values are expressed as mean difference (I-J) ± SE. Bold values indicate statistical significance at the *p* < 0.05 level.

## 4 Discussion

In this study, we examined the effects of transcranial direct current stimulation (tDCS) and transcranial alternating current stimulation (tACS), each combined with cognitive-motor dual-task training (CMDT), on cognitive function, physical performance, and activities of daily living in subacute stroke survivors. The findings demonstrated that dual-target stimulation protocols simultaneously targeting the affected primary motor cortex (M1) and the left dorsolateral prefrontal cortex (DLPFC), in combination with CMDT, produced superior outcomes compared to sham stimulation. Specifically, the tDCS group exhibited significantly greater improvements in cognitive performance (VCAT) and physical function (Fugl-Meyer Lower Limb, Berg Balance Scale) compared to the sham group, and superior motor gains (FMA-LE) relative to tACS. Both active stimulation groups (tDCS and tACS) showed comparable benefits over the sham group in enhancing dual-task mobility (TUG-Dual) and reducing depressive symptoms (HAMD). Although significant interactions were observed for executive function measures (TMT-A and TMT-B) and single-task walking (TUG-single), post-hoc analyses revealed no significant between-group differences after correction.

The significant improvement in VCAT scores in the tDCS + CMDT group compared to sham suggests enhanced visual cognitive processing, attributable to increased DLPFC excitability. The DLPFC is a core area for advanced cognition, especially for working memory and attention. tDCS enhances the excitability of the DLPFC, improving patients’ processing ability of cognitive elements, and thus demonstrating a significant advantage on the VCAT, which are aligned with the concept of “primed learning,” where non-invasive neuromodulation enhances the excitability and plasticity of targeted brain regions, optimizing them for subsequent training (38, 39). The DLPFC, a core region in executive control and attentional processing, and M1, central to motor control and recovery, were both modulated through anodal stimulation. This aligns with the “state-dependent” model of neurostimulation, where the effectiveness of behavioral interventions is amplified when the brain is preconditioned to a more plastic or excitable state (40). Stroke survivors often rely on compensatory cognitive strategies for motor deficits, and DLPFC priming could alleviate this burden (41, 42).

The motor function improvements observed (FMA-LE and BBS) in the tDCS + CMDT group can be attributed to enhanced M1 excitability and reorganization, which are essential for balance, gait, and coordinated movement. Reduced excitability in the affected M1 has been linked to motor dysfunction post-stroke (43), and tDCS is known to upregulate M1 plasticity, facilitating implicit motor learning and balance control. Importantly, the DLPFC and M1 are not only anatomically connected, but also engage in bidirectional functional communication that modulates motor behavior (44). Dysregulation of this circuitry, particularly excessive inhibitory control of M1 by DLPFC, may impair recovery. tDCS may help rebalance this inhibitory interaction, enhancing motor output and overall function. Anodal tDCS applies a continuous direct current to the cortex, inducing a sustained depolarization of the neuronal resting membrane potential beneath the anode. This action increases neuronal excitability and spontaneous firing rates. This effect is widely accepted as inducing Long-Term Potentiation (45) (LTP)-like plasticity, which promotes stable and long-lasting facilitation of M1. Indicators directly related to limb motor control and postural stability, such as FMA-LE and BBS, are highly sensitive to this M1 excitability enhancement.

While tDCS demonstrated superior effects in driving local M1 plasticity for motor function and balance, our findings highlight the unique role of tACS as an effective network modulator, particularly when applied at the Gamma frequency over the DLPFC. This specialized approach yielded treatment effects that were equivalent to tDCS and significantly superior to Sham in complex domains like dual-task performance and mood regulation. The observation that the tACS group achieved significant improvement in the TUG-Dual task vs. Sham, with efficacy comparable to the tDCS group, reveals the potent capability of tACS in synchronizing functional brain networks. Gamma oscillations (typically >30 Hz) are widely recognized as the fundamental neural substrate for high-level cognitive functions, including information binding, working memory, selective attention, and executive control (46). By applying tACS at the Gamma frequency over the DLPFC, the external current is hypothesized to resonate with, or entrain, the inherent Gamma activity of the DLPFC (47). This entrainment enhances the synchronization of neural firing within the frontal regions. This enhancement in Gamma rhythm synchronization significantly improves the speed and efficiency of information transfer between the DLPFC and the motor planning/execution areas. CMDT challenges the ability to coordinate motor and cognitive demands often impaired in stroke survivors due to deficits in executive control and reduced reserve capacity (41). For dual-task performance (TUG-CMDT), both tDCS and tACS outperformed sham, suggesting effective network synchronization. During the TUG-Dual task, the patient must efficiently allocate resources between ambulation (motor task) and the cognitive load. tACS-optimized Gamma synchronization ensures a more streamlined information flow, thereby mitigating the cognitive-motor interference often experienced in stroke, leading to comparable clinical gains with tDCS. This reflects a lower cognitive load and more automatic integration of tasks post-intervention, likely mediated by DLPFC-M1 connectivity (44). Additionally, DLPFC stimulation may have directly contributed to the observed reduction in depressive symptoms (HAMD), given its role in emotion regulation and its known connections with limbic structures (38).

Furthermore, the significant reduction in depressive symptoms (HAMD scores) observed in the tDCS and tACS groups may reflect both indirect effects of improved cognitive and physical function, creating a positive feedback loop between mood and performance (48), as well as the direct neuromodulatory influence of DLPFC stimulation on emotion regulation circuits (38). The DLPFC is a critical hub not only for executive function but also for the modulation of affective states (49). Overall, this study highlights the interconnectedness of cognitive, motor, and emotional recovery after stroke, and demonstrates how dual-site tDCS can serve as an effective neural priming tool to enhance the benefits of CMDT. The coordinated stimulation of M1 and DLPFC likely promoted widespread neuroplastic changes, including increased intercortical connectivity and efficiency of functional networks, ultimately improving the brain’s capacity to recover from stroke-related deficits (44).

Improvements in cognitive and behavioral performance following tES over M1 have been linked to increased cortical plasticity, hypothesized to occur through a reduction in *γ*-aminobutyric acid (GABA) concentrations, an inhibitory neurotransmitter critical to motor learning processes (50). Concurrently, superior cognitive outcomes after DLPFC stimulation are likely due to increased excitability in the executive control and ventral attention networks (51). On a molecular level, tDCS has been shown to increase brain-derived neurotrophic factor (BDNF) expression, dendritic spine density in peri-infarct M1, and enhance connectivity between motor and somatosensory cortices (52, 53). Anodal stimulation may also upregulate MAP-2 and GAP-43 expression, promoting dendritic plasticity and synaptogenesis in the ischemic penumbra and contralateral cortex processes crucial for neurorepair (50). Animal studies (54) further suggest that tDCS exerts neuroprotective and regenerative effects via mechanisms such as reducing glutamate excitotoxicity, neuroinflammation, and oxidative stress, while promoting angiogenesis, neurogenesis, and neurotransmitter regulation. In the subacute phase of stroke, these mechanisms are especially potent.

Simultaneous anodal stimulation of the left DLPFC, a key hub of the Central Executive Network (CEN), likely enhanced cognitive control across brain networks. The DLPFC’s intrinsic connectivity within the CEN and its regulatory role over the Default Mode Network (DMN) and Salience Network (SN) are well-established (55). Strengthened DLPFC function may improve attentional control, reduce distractibility, and facilitate more efficient working memory updating (56). These cognitive improvements are reflected in cognitive scales scores in the present study. Such neurophysiological changes suggest enhanced top-down modulation and more efficient processing of task-relevant information. Overall, these adaptations support better performance in complex cognitive-motor tasks like dual-task walking, highlighting the translational potential of DLPFC-targeted stimulation combined with cognitive-motor training.

Both modalities were well-tolerated, with mild headaches in two tDCS participants leading to discontinuation, aligning with low adverse event rates in literature (57).

### 4.1 Study limitations

Despite promising results, limitations include the modest sample size (n = 69), potentially limiting generalizability across stroke etiologies (ischemic and hemorrhagic were pooled). The wide inclusion window resulted in baseline imbalances in onset subgroups, though overall comparability was maintained. Multiple endpoints raise Type I error risk, necessitating validation in larger trials. The 3-week duration precludes insights into long-term retention, and absence of neuroimaging (e.g., fMRI, DTI) limits mechanistic elucidation. Future studies should stratify by stroke type, incorporate lesion metrics, and extend follow-up.

### 4.2 Clinical implication

These findings suggest dual-site tDCS + CMDT as a scalable, non-invasive adjunct for subacute stroke rehabilitation, targeting executive dysfunction that impairs independence. tACS offers comparable benefits in dual-task and mood domains, providing options based on patient needs. Optimizing parameters (e.g., intensity, duration) and personalizing via predictors could enhance outcomes.

## 5 Conclusion

In summary, this study demonstrates that dual-site anodal tDCS and tACS targeting M1 and left DLPFC, when combined with CMDT, are safe and effective strategies for enhancing recovery after stroke. Dual-site tDCS produced superior improvements in motor function, balance, and cognition, while tACS showed comparable benefits in dual-task performance and mood regulation. Overall, tDCS offers stronger facilitation of local motor plasticity, whereas tACS effectively supports network-level cognitive and emotional recovery, underscoring the therapeutic potential of both modalities in post-stroke neurorehabilitation.

## Data Availability

The de-identified participant data (including data dictionary), statistical codes, and other research materials can be obtained upon reasonable request to the corresponding authors. Approval requires the signing of a data access agreement and compliance with the requirements of the ethics committee.
