# Supplementary material for: Comparative effects of transcranial direct and alternating current stimulation combined with cognitive-motor dual-task training on functional and cognitive recovery in stroke survivors
Source: Front Neurol. 2026 Feb 6;17:1720429. doi: 10.3389/fneur.2026.1720429 (PMC12921704; doi:10.3389/fneur.2026.1720429)
Supplement: Supplementary file 1 [file Data_Sheet_1.PDF]

Supplementary Material

After the baseline assessment, all participants were randomly assigned to three groups: tDCS with DT; tACS with DT; sham stimulation with DT using stratified block randomization based on the number of stroke survivors using the CREATE A RANDOMISATION LIST with block size of 6 (Stroke survivors was stratified by age, gender and then randomized 1:1:1 to sequential exercise. Stratification factors: Age group (40-75) and gender (female, male) in Table 1.

Table 1 Randomization Process

**Block Identifier**	**Sequence within Block**	**Treatment Category**	**Age Group Stratum**	**Treatment Assigned**
1	1	3	40-75	Sham stimulation with DT
1	2	2	40-75	tACS with DT
1	4	3	40-75	Sham stimulation with DT
1	5	2	40-75	tACS with DT
1	6	1	40-75	tDCS with DT
2	1	1	40-75	tDCS with DT
2	2	1	40-75	tDCS with DT
2	3	2	40-75	tACS with DT
2	4	3	40-75	Sham stimulation with DT
2	5	3	40-75	Sham stimulation with DT
2	6	2	40-75	tACS with DT
3	1	1	40-75	tDCS with DT
3	2	2	40-75	tACS with DT
3	3	3	40-75	Sham stimulation with DT
3	4	3	40-75	Sham stimulation with DT
3	5	1	40-75	tDCS with DT
3	6	2	40-75	tACS with DT
4	1	2	40-75	tACS with DT
4	2	3	40-75	Sham stimulation with DT
4	3	3	40-75	Sham stimulation with DT
4	4	1	40-75	tDCS with DT
4	5	1	40-75	tDCS with DT

4	6	2	40-75	tACS with DT
5	1	3	40-75	Sham stimulation with DT
5	2	2	40-75	tACS with DT
5	3	3	40-75	Sham stimulation with DT
5	4	1	40-75	tDCS with DT
5	5	2	40-75	tACS with DT
5	6	1	40-75	tDCS with DT
6	1	1	40-75	tDCS with DT
6	2	3	40-75	Sham stimulation with DT
6	3	3	40-75	Sham stimulation with DT
6	4	2	40-75	tACS with DT
6	5	1	40-75	tDCS with DT
6	6	2	40-75	tACS with DT
7	1	2	40-75	tACS with DT
7	2	1	40-75	tDCS with DT
7	3	3	40-75	Sham stimulation with DT
7	4	3	40-75	Sham stimulation with DT
7	5	1	40-75	tDCS with DT
7	6	2	40-75	tACS with DT
8	1	2	40-75	tACS with DT
8	2	2	40-75	tACS with DT
8	3	3	40-75	Sham stimulation with DT
8	4	1	40-75	tDCS with DT
8	5	1	40-75	tDCS with DT
8	6	3	40-75	Sham stimulation with DT
9	1	1	40-75	tDCS with DT
9	2	2	40-75	tACS with DT
9	3	3	40-75	Sham stimulation with DT
9	4	2	40-75	tACS with DT

9	5	3	40-75	Sham stimulation with DT
9	6	1	40-75	tDCS with DT
10	1	1	40-75	tDCS with DT
10	2	2	40-75	tACS with DT
10	3	1	40-75	tDCS with DT
10	4	3	40-75	Sham stimulation with DT
10	5	2	40-75	tACS with DT
10	6	3	40-75	Sham stimulation with DT
11	1	1	40-75	tDCS with DT
11	2	2	40-75	tACS with DT
11	3	3	40-75	Sham stimulation with DT
11	4	2	40-75	tACS with DT
11	5	1	40-75	tDCS with DT
11	6	3	40-75	Sham stimulation with DT
12	1	3	40-75	Sham stimulation with DT
12	2	2	40-75	tACS with DT
12	3	3	40-75	Sham stimulation with DT
12	4	3	40-75	Sham stimulation with DT
12	5	1	40-75	tDCS with DT
12	6	2	40-75	tACS with DT

Section2 Dual Task Protocal

a. Dual Task Training Method

An evidence-based DTT program for stroke survivors was not available (Han et al. 2024). The training content was selected according to the DTT manual, which was designed by experienced physical therapists in the Rehabilitation Department of Medicine, Second Affiliated Hospital of Kunming Medical University, and was based on previous literature. The physical therapist adjusted the training difficulty level according to the participant's performance. Thus, we developed a DTT program based on the combination of the available evidence (Costa-Ribeiro et al. 2021) and experience of senior therapists in the department. Primary motor training was

conducted using activities with three levels of difficulty. The components of DTT include gait training, balance, and flexibility, which address the functional impairment of stroke survivors, such as slow motor performance, walking impairment, and low physical activity (Aguiar et al. 2020; Kim 2022). Participants evolved from one level to the next when performance on the previous level was free of errors. The second cognitive tasks were decided by the results of cognitive assessment scales. It was noteworthy that these interventions were individually tailored. The domains of cognition, namely short-term memory, attention, calculation, auditory discrimination, reasoning and logic, and naming objects, were selected for cognitive training in our present intervention. This selection was based on the existing literature among stroke survivors with cognitive impairment (Huang et al. 2022; Zhang et al. 2023).

Each session was divided into 5 min of warm-up exercises in Table 2 and 30 minutes motor exercises simultaneously with cognitive exercises in Table 3 and Table 4. At the end, participants were asked to report the functional difficulties experienced during DTT.

Table 1 Exercises for warming-up

Items	Frequency
Slow marching and swinging arms	12 times
Side Wall Push	5 times Left + 5 times Right
Trunk Lateral stretch	20 s × 5 times Left + 20 s × 5 times Right
Trunk and head rotation stretch	20 s × 5 times Left + 20 s × 5 times Right
Calf muscle stretch	20 s × 5 times Left + 20 s × 5 times Right
Thigh muscle stretch	20 s × 5 times Left + 20 s × 5 times Right
Gluteal muscle stretch	20 s × 5 times Left + 20 s × 5 times Right
Hamstrings muscle stretch	20 s × 5 times Left + 20 s × 5 times Right
Chin Forward / Backward	10 times Forward / 10 times Backward
Mountain Pole	10 times
Stop Horse	10 times
Stretch both upper limbs at different angles	10 times
Bend and stretch with your hands on your head	10 times

Table 2 Primary motor training

Tasks	Level 1	Level 2	Level 3
Standing Balance Station (no hand hold allowed)			
Standing (30s)	Feet together (eyes open/eyes closed)	Half Tandem/Full tandem (eyes open/eyes closed)	Change support (surface balance pad, wobble board)
	Progression criterion: Feet together eyes closed standing without minimal postural sway, no handhold or supervision	Single leg standing with other leg on edge of step eyes close without minimal postural sway, no handhold or supervision.	Eyes closed, feet together standing on 4 cm balance pad without minimal postural sway, no handhold or supervision.
Weight-shifting(30s)	Inter-trial variability: Forward-Backward, laterally	Narrower BOS	Change support surface and speed
	Feet shoulder width do forward/ lateral reaching and reach 12cm/7cm far away for each time as quickly as possible	Feet together do forward/lateral reaching and reach18m /10cm futher each time	Feet together on balance pad do forward/lateral reaching 6times and reach 15cm /8cm further each time
Mobility Station (walking aid only if necessary)			
Walking in circle(30s)	Around a big circle.	Change speed	Change support surface
	Walking forward around a square table (l=60 cm) ,Pathway is 12inches = 30.48cm ,Cannot deviate out of pathway .	Same situation as level one and finishing 5 circles within 30s.	Walk forward on foam around square table and cannot deviate out of pathway, 5 circles within 30sec
Stepper (30s)	Forward-turn around –forward back/laterally	Increase the height, try as fast as possible	Do it with softer surface
	Finish forward-turn around -forward back 3 times /laterally stepper 4 times within 30s(7 inches height stepper)	Can finish forward-turn around -forward back 5 times / laterally stepper 6 times within 30s(20inches stepper).	Use a 20inches stepper with a balance pad and can finish forward-turn around -forward back 8 times/ laterally stepper 9 times .
Walking with Obstacle Avoidance (30s)	The distance between obstacles wide	Narrow the distance, Try as fast as possible	inter-trial variability: Forward, Laterally, Backward
	Forward walking for 4 obstacles within 30s (distance between each is1.5m)	Forward walking for 12 obstacles within 30s (distance between each obstacle is 1 meter)	Backwards walking for 20 obstacles within 30s (distance between each obstacle are 1 meter)
Walking with Rope ladder	Stepping one foot through each rung. Both feet in each space (alternating lead foot)	Inter-trial variability: (Forward, Laterally) Try as fast as possible	Various separation

(30s)	Forward walking for 16 rungs within 30s (distance between each rungs is 0.5metre)	Forward --laterally walking for 24 rungs within 30s (distance between each rung is 0.3metre)	Forward walking for 32 rungs within 30s (each obstacle are between like 0.3-0.6-0.3-0.6 meter)

Table 3 Secondary cognitive training

Secondary Cognitive Task	Description			
Naming Objects	Subjects are asked to name objects that belong to the same category (For example, Color, shape, flower, body part / Organs, drinks, daily necessities, occupations, festivals, sports, nationalities, vegetables, names of relatives, animals, public facilities, kitchen supplies, place names, nature, Yunnan tourist attractions, places of interest and historic sites, historical celebrities, electrical appliances, washing supplies, hospitals, stationery, book titles, kitchen supplies, materials, cars or clothing brands, shoes, universities, emotions, idioms, disciplines, Musical Instruments , Shop)			
Auditory Discrimination	Subjects are asked to identify the voices/noises from a compact disc (20 animals, 20 Musical Instruments, 20 sports, 20 daily necessities, 20 nature and transportation)			
Counting Forward	1+1=<br>7-1=<br>9+2=<br>9-3=	88-3=<br>94-8=<br>32-6=<br>54-8=	76-2=<br>67-3=<br>35-1=<br>58-4=	86-23=<br>65+24=<br>36+24=<br>87-31=
Remembering Items	Participants are asked to memorize a multiple-item shopping list：<br>1, cake, battery, lamp, Chinese medicine, watch, shoes, rehabilitation department<br>2, milk, powder, tape, scissors, tofu, phone, right foot, juice<br>3, police, toothbrush, watch, milk, battery, tea, plum<br>4, slippers, police, mother, phone, tofu, white, doctor<br>5, rose, left hand, milk, toothbrush, Mid-Autumn Festival, leather shoes, rehabilitation, department			
Answering Questions	The correct answer will be received in advance from the participant's family or caregiver.<br>1. What is your child's name? What's your grandson's name? What are your siblings' names?<br>2. When is your child's birthday? Do you remember your grandson's birthday? Do you remember the birthdays of your siblings?<br>3. When is your birthday and your spouse's date of birth?<br>4. What is your height? How much do you weigh? Where do you live?<br>5. What exercises did you do after your stroke?			
Telling Story	Participants are asked to tell the exercise instructor any story:<br>1. What is your hobby and why do you like it?<br>2. What is your favorite place and why?<br>3. What's your favorite food and why?<br>4. What's your favorite TV show and why?<br>5. What did you have for lunch? What are their characteristics?			

a. Dual-task training progression metrics

The DTT program was delivered using a structured, progressive, and individualized protocol designed to simultaneously challenge motor and cognitive domains. Training difficulty was advanced in a stepwise manner, with participants progressing to the next level only after completing the current level without performance errors, compensatory movements, or loss of balance, thereby ensuring appropriate task difficulty and sustained engagement within individual capability ranges. The motor component included standing balance, weight-shifting, stepping, and mobility tasks, with progression systematically manipulated across four parameters: base of support (from wide stance to feet together or tandem stance), visual condition (eyes open to eyes closed), support surface stability (firm ground to balance pad or wobble board), and movement complexity and speed (e.g., increased reach distance, reduced obstacle spacing, and multidirectional movement). For example, participants progressed from static standing balance on stable surfaces with eyes open to dynamic tasks performed on unstable surfaces with eyes closed, and from walking with widely spaced obstacles on firm ground to navigating narrow obstacle spacing or compliant surfaces. Concurrently, cognitive tasks—including naming, auditory discrimination, simple arithmetic, memory recall, and storytelling—were performed during motor activities, with cognitive load adjusted by increasing stimulus quantity, reducing response time, or integrating multimodal stimuli. Training progression was quantitatively monitored using motor accuracy (successful trials without postural deviation), cognitive accuracy (% correct responses), task completion time (s) during standardized 30-second trials, and the highest level achieved (Level 1: basic; Level 2: intermediate; Level 3: advanced). Therapists continuously supervised performance to ensure safety, providing minimal verbal cues without physical assistance. Advancement to higher difficulty levels occurred when participants demonstrated stable performance, defined as ≥90% task accuracy and absence of postural instability, allowing for individualized progression while balancing challenge and safety and facilitating motor–cognitive integration.

In this study, the TMT Parts A and B were administered using the standard paper-and-pencil format in Mandarin Chinese.

Primary outcome in TMT is completion time (in seconds)

Secondary outcome is accuracy/number of errors.

We only reported completion time, because it is the most sensitive and classic indicator of processing speed, visual search, attention, and executive function/task-switching abilities.

TMT- A consisted of 25 encircled numbers (1–25) randomly distributed on a sheet of paper, requiring participants to connect them in ascending numerical order as quickly and accurately as possible.

For TMT-B, we employed a commonly used Chinese adaptation that substitutes letters with black and white circles (e.g., connect 1-black → 1-white → 2-black → 2-white, etc.)

TUG test was performed on a marked 3-meter walkway in a quiet, well-lit room. Participants were seated in a standard armless chair (seat height approximately 46 cm) with their back against the chair. Standardized instructions were provided: "On the word 'go', stand up, walk at your comfortable pace to the mark on the floor 3 meters away, turn around, walk back, and sit down again." Timing began on the word "go" and stopped when the participant's buttocks touched the seat.

Single-task TUG: Participants performed the task without any additional cognitive load. The outcome was the time (in seconds) to complete the task. Three trials were conducted, and the average time was used for analysis.

Dual-task TUG (cognitive-motor dual-task): Participants performed the same motor sequence while simultaneously performing a cognitive task (serial subtraction by 3s starting from a random number between 90 and 100, or counting backwards aloud). The cognitive task began on the word "go" and continued until the participant sat down.
